# Principles and Applications of CRISPR Toolkit in Virus Manipulation, Diagnosis, and Virus-Host Interactions

**DOI:** 10.3390/cells11060999

**Published:** 2022-03-15

**Authors:** Saleh Jamehdor, Sara Pajouhanfar, Sadaf Saba, Georges Uzan, Ali Teimoori, Sina Naserian

**Affiliations:** 1 Cellular and Molecular Research Center, Zahedan University of Medical Sciences, Zahedan 989155432609, Iran; salehjamehdor@alumni.ut.ac.ir; 2 Department of Pediatrics, Washington University School of Medicine, St. Louis, MO 63110, USA; pajouhanfar.s@wustl.edu; 3 Center for Molecular Medicine & Genetics, Wayne State University School of Medicine, Detroit, MI 48201, USA; ssaba@med.wayne.edu; 4 INSERM UMR-S-MD 1197, Hôpital Paul Brousse, 94800 Villejuif, France; georges.uzan@inserm.fr; 5 Paris-Saclay University, 94800 Villejuif, France; 6 Department of Virology, Faculty of Medicine, Hamadan University of Medical Sciences, Hamadan 6517838738, Iran; 7 CellMedEx, 94100 Saint Maur Des Fossés, France

**Keywords:** virus, CRISPR, genetic engineering, SARS-CoV-2, HIV-1

## Abstract

Viruses are one of the most important concerns for human health, and overcoming viral infections is a worldwide challenge. However, researchers have been trying to manipulate viral genomes to overcome various disorders, including cancer, for vaccine development purposes. CRISPR (clustered regularly interspaced short palindromic repeats) is becoming one of the most functional and widely used tools for RNA and DNA manipulation in multiple organisms. This approach has provided an unprecedented opportunity for creating simple, inexpensive, specific, targeted, accurate, and practical manipulations of viruses, such as severe acute respiratory syndrome coronavirus 2 (SARS-CoV-2), human immunodeficiency virus-1 (HIV-1), and vaccinia virus. Furthermore, this method can be used to make an effective and precise diagnosis of viral infections. Nevertheless, a valid and scientifically designed CRISPR system is critical to make more effective and accurate changes in viruses. In this review, we have focused on the best and the most effective ways to design sgRNA, gene knock-in(s), and gene knock-out(s) for virus-targeted manipulation. Furthermore, we have emphasized the application of CRISPR technology in virus diagnosis and in finding significant genes involved in virus-host interactions.

## 1. Introduction

The diversity of viral genome organizations and their replication systems is the most remarkable aspect of viruses’ molecular biology. In general, viruses are classified into two major groups: DNA or RNA viruses. According to David Baltimore’s comprehensive classification in 1971, viruses are divided into seven groups [1]. RNA-containing viruses have single or double-strand RNAs that can be replicated through one of the two unique molecular pathways, either RNA-dependent RNA synthesis (RNA replication) or RNA-dependent DNA synthesis (reverse transcription) as in the retroviruses (e.g., HIV), which is followed by DNA replication and transcription. The latter cellular transcription machinery produces viral genomic RNA, as well as host mRNAs. Among single-stranded RNA viruses, three forms of viral RNA have been identified in infected cells. These include single-stranded RNA, replicative intermediate (RI) RNA, and replicative type (RF) RNA. Despite the fact that DNA-containing viruses can be single- or double-stranded, their DNA replication mechanism is similar to other organisms, whether it is through the cellular or viral DNA replication machinery. Except for poxviruses, all DNA viruses use cellular transcription machinery (cellular RNA polymerase II and III for transcription). Viruses with RNA genomes replicate entirely in the cytoplasm, except for influenza viruses and retroviruses (e.g., HIV), for which replication takes place in the nucleus. These viruses are less associated with the cell nucleus compartments. On the contrary, most DNA viruses replicate within the nucleus, except for poxviruses, which replicate entirely/fully in the host cell cytoplasm [2].

The advances in different genome-editing techniques within the last two decades have revolutionized biology. Among them, zinc finger nuclease (ZFN) and transcription activator-like effector nucleases (TALEN) are the first introduced techniques (Table 1) [3,4]. However, the development of CRISPR in 2012 was a turning point in this field [5]. After the discovery of the CRISPR technology, the majority of research studies in all organisms (especially in viruses) have been based on this technique. This popularity can be attributed to the inexpensiveness, simplicity, functionality, and accuracy of this method to edit the genomes of various organisms [6]. The CRISPR technique is derived from the adaptive immune system of bacteria, consisting of an endonuclease protein called Cas9 that is specific and functional thanks to two RNAs called the CRISPR RNA (crRNA). It has obtained its enzyme functionality by attaching to the trans-activating crRNA (tracrRNA), which operates with high precision. Most studies on this system have used Cas9 (CRISPR-associated protein 9) obtained from *Streptococcus pyogenes*. In the optimized version of this technique, an RNA called single-guide RNA (sgRNA) is designed to have 20 nucleotides complementary to edit the target site, through which it modifies various organisms’ genomes. The specificity of this technique is determined by these 20 nucleotides and a sequence called protospacer-adjacent motif (PAM). The PAM sequence is located right before the 20 nucleotides mentioned earlier and can be different for each Cas9s derived from various bacteria [5,7]. For example, regarding *Streptococcus pyogenes* Cas9 (spCas9), this sequence is “5′-NGG-3′” and for (*Staphylococcus aureus* Cas9 (saCas9), this sequence is “5′-NNGRRT-3′”, which is essential for having a functional system [8,9]. Following this sgRNA, there are several stem-loop structures to make RNA-mediated conformation activation of Cas9 and DNA cleaved three nucleotides after the PAM [5,7] either through nonhomologous end joining (NHEJ) or homology-directed repair (HDR). The contribution of each method (NHEJ and HDR) varies in different organisms. In human cells, NHEJ repair occurs more than HDR. In the NHEJ repair method, which is used to knock-out (KO) genes, one or more nucleotides are added to or are removed from the cut site (indel mutation) where the binding of double-stranded DNA occurs. In the HDR system, we need a template DNA that is complementary to the two ends of the cleaved region with the insertion nucleotides present in the middle regions of this fragment [10,11,12]. This DNA fragment is also inserted into the target cell along with the CRISPR system. This method is used to knock-in (KI) into the cut region [13].

**Figure 1:**
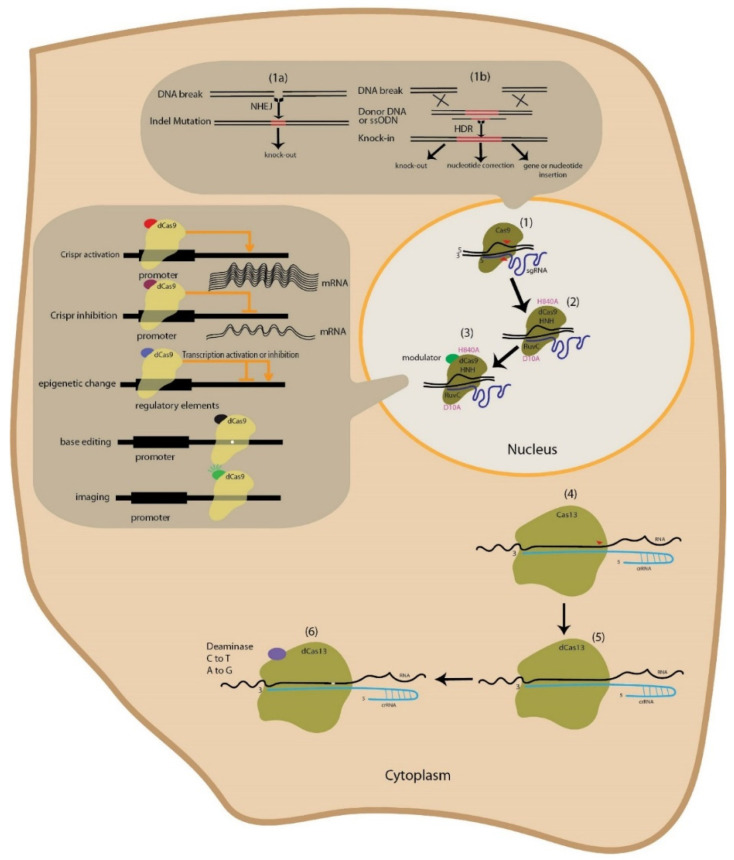
An overview of the CRISPR technology. (1) In the CRISPR system, Cas9 target sites are identified by 20 sgRNA nucleotides that complement the target site. Then, double-strand DNA blunt end cuts are obtained using the Cas9 protein. After DNA is cut within the cell, DNA repair can be accomplished either through NHEJ or HDR. (1a) In NHEJ, the predominant repair system in mammalian cells, a few nucleotides are added to or removed from the cut area (INDEL mutation). This repair system is mainly used for gene knock-out. (1b) The repair system used by cells, the HDR, is performed with high accuracy using complementary arms of the incised area. The scientists have inserted several nucleotides into the cut region by inserting ssODN, which has short arms. Larger arms (from a few hundred bases to several kilobases) are used to insert larger fragments that complement the target position. This method is used for gene knock-out, nucleotide correction, and gene or nucleotide insertion. (2) By creating point mutations in the HNH (H840A) and RuvC (D10A) domains, a deactivated Cas9 is created that is attached only to the target area, incapable of making cuts. (3) Fusion of different modulators including transcription activators and inhibitors, epigenetic modifiers, and base editing affect gene expression or the reporter domains, which, for imaging purposes, can be used to make targeted changes at the transcriptional level and to purposefully label an area of DNA. (4) Additionally, Cas13 can make targeted RNA cleavage (guided by crRNA). (5) By creating a point mutation in the Cas13 protein-coding sequence, Cas13 is deactivated with the ability to bind to RNA without cutting it. (6) By attaching the deaminase domains, the ability to make single-base changes in RNA is obtained.

One of the drawbacks of the CRISPR system is being off-target; it may mistakenly identify other regions and cause unintended changes [14]. By modifying Cas9 (modifying D10A (aspartic acid 10 alanine) and H840A (histidine 840 alanine)) in this protein), new features have been added to the protein, such as making it incapable of cutting DNA strands, or, with one mutation, it can cut one DNA strand [15]. Each gRNA individually recruits Cas9-D10A, resulting in nicking both DNA strands in the nearby locations and, in consequence, creating a double-strand break (DSB) at the intended target site [7]. This system uses two sgRNAs designed for the upper and lower parts of the DNA strand. By using Cas9, which cuts only one strand of the double-stranded DNA (Cas9 nickase) and designs that lead to two cuts with a specified distance, a double-strand break is generated. This method has helped to enhance the accuracy of the system and reduce its off-target probability by 1500 times compared to the wild-type spCas9 [15,16]. To overcome off-target effects, a lot of research is being done to change the sgRNA structure and Cas protein engineering to increase their accuracy and reduce PAM dependency [17]. The use of temporal activators by optogenetic technology (photoactivable Cas9) and induction systems by chemical components are applicable. This technology allows us to activate and deactivate it whenever required, which causes a off-target reduction in off-target effects [18,19]. Additionally, the accurate design of sgRNAs with bioinformatic tools that have the least off-target effects is another possible method [20]. It has been shown that if the number of sgRNA nucleotides, which are attached to the target site, is reduced to 17, the probability of an off-target effect occurring is significantly decreased [21]. The ability to modify gene transcription expression and epigenetics is also obtained by binding modulators that change the transcription regulation to Cas9 and using inactive Cas9 (Figure 1) (Cas9 without cutting function) [22,23,24,25,26,27]. Researchers have developed the ability to make changes and identify nucleic acids by attaching transcription activators, inhibitors or epigenetic modifiers (i.e., acetylation or deacetylation, methylation or demethylation), or by attaching markers such as GFP (green fluorescence protein) base editors. Prior to the discovery of CRISPR technology, it was not possible to make these changes with high accuracy and specificity, but the identification of this technology has greatly helped to understand molecular activities such as virus host-cell interaction. The delivery of these systems into the cell is one of the most important components in making effective changes. Additionally, using sgRNAs with high efficiency and correctly targeting the desired locations would be of great help to make this technology more practical [28,29,30]. We can also make changes to RNA by using deactivated Cas13 and by its attachment to base editors and epigenetic modifiers [31].

In 2015, a new variant of the system, called CRISPR/Cpf1 (CRISPR-associated endonuclease in *Prevotella* and *Francisella 1*), was discovered. In this system, the sgRNA and Cpf1 protein (Cas12a) is smaller than the CRISPR/Cas9 system, and there is only one stem-loop at the end of the sgRNA. However, unlike Cas9, which produces a blunt end, the Cas12a protein provides a sticky end with a sequence of 24 nucleotides complementary to the target site. Additionally, the Cas12a PAM sequence is AT-rich (unlike the Cas9 PAM sequence, which is GC-rich) [32]. This system is not functional in some viruses. For example, the herpes simplex virus (HSV) does not work/function in CRISPR/Cas12a due to its high GC content [33]. A comparison of CRISPR application systems (CRISPR/Cas9, CRISPR/Cas12, and CRISPR/Cas13) is provided in Table 1. With the use of this technology, scientists could perform fast and effective nucleotide editing, gene knock-in and gene knock-out in viruses, but the efficiency of these changes is different in various viruses and articles. CRISPR technology can work on all organisms that have RNA and DNA genomes. The identification and treatment of diseases are some of the most important applications for this method. This technology is also used as a tool to identify genes and pathways associated with diseases [34]. Although off-target effects can be considered a potential disadvantage, in some cases, it can also be considered as an advantage to produce more mutations in viruses [35].

In this review, we have focused on these problems and provided solutions to overcome them. Furthermore, we discuss the latest pieces of information on the application of this technology in virus-cell interaction identification and CRISPR technology application in virus diagnosis.

**Table 1:** Comparison of the CRISPR/Cas9, CRISPR/Cas12, and CRISPR/Cas13 toolkits [36,37,38,39,40].

CRISPR Toolkit	CRISPR/Cas9	CRISPR/Cas12a	CRISPR/Cas13
sgRNA (Cas9) or crRNA (Cas12 and 13)	Special sgRNA for attaching to a specific site on DNA	Special crRNA for attaching to a particular site on DNA	Special crRNA for attaching to a particular site on RNA
Cut domain(s)	Ruv C- and HNH-like endonuclease domains	Ruv C-like endonuclease domain	HEPN domains
Specificity	High	High	High
Vectors	Available with low cost (usually)	Available with low cost (usually)	Available with low cost (usually)
PAM	GC-rich	AT-rich	Diverse for various systems, except for Cas13d, which does not have this sequence
Origin	Prokaryotes	Prokaryotes	Prokaryotes
Methylated DNA	Lack of binding to methylated cytosine	Not detected	Not studied
Multiplexing	Easy and functional	Easy and functional	Easy and functional
Number of specific complementary nucleotides to the target region	18–24 nucleotides	23–25 nucleotides	22–30 nucleotides
Break	Double-strand DNA	Double-strand DNA	Single-strand RNA
Cut	Blunt end	Sticky end	Not studied

## 2. Principles of sgRNA Design

**Figure 2:**
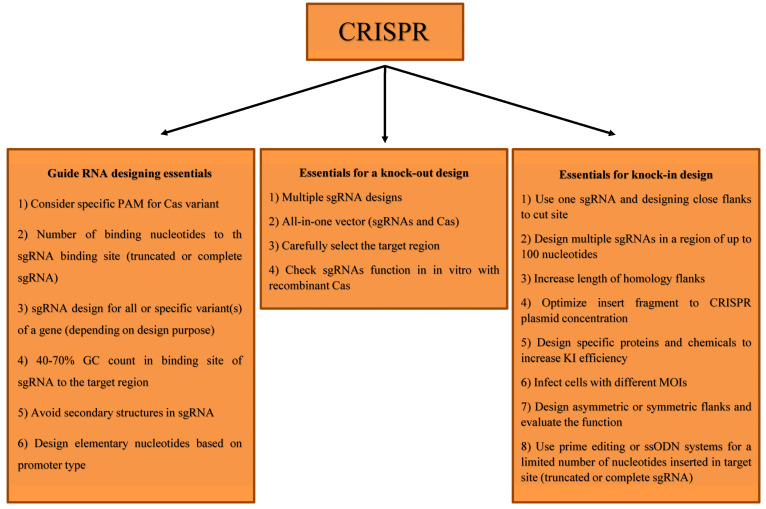
Essentials for optimum knock-out, knock-in, and gRNA design.

sgRNA is a single RNA with stem loop(s) and custom-designed nucleotides to facilitate attachments to the target region; in fact, Cas9-targeted endonuclease recognizes targeted sequences with sgRNA. The first and key step for having an effective and efficient system is a proper/accurate sgRNA design. The importance of this design is that any error affecting the system’s performance results in a lack of conclusions at later stages [41]. Several well-known and consistent rules for this design are important for this system’s performance in all organisms. First of all, the system’s PAM sequence should be considered throughout the design process [5,7]. The other aspect to consider is the number of PAM nucleotides and nucleotide sequences; as mentioned in the introduction section, various Cas9s have been identified in different bacteria, each with their particular PAM. Thus, depending on the origin of Cas9, PAM (both in terms of nucleotide number and nucleotide sequence) could be different. The accuracy of the system increases with the number of PAM nucleotides. However, the number of sites designed to be targeted will be reduced [42,43]. The first 10 to 12 nucleotides (next to the PAM sequence) of the 20 nucleotides designed in the CRISPR/Cas9 system that is complementary to the target site is called the seed sequence, and any mismatch in this region leads to system malfunction. However, 3–5 mismatches in the next 8–10 nucleotides are tolerable [44]. It is recommended that the designed region on sgRNA be sequenced to confirm that there are no SNPs that could cause system deactivation. It has been shown that if the sgRNA length is reduced to 17 nucleotides (truncated sgRNA), it does not disrupt the system performance and also increases the system specificity, leading to decreased off-target effects elsewhere in the genome [45]. It should be noted that the system operates highly specifically given the small and specific genomic sequences in many viruses. Therefore, off-targeting genome engineering of viruses with the CRISPR system would not have any adverse effect in many cases. However, in instances where we want to design in repeated sequences of the viral genome, this approach can reduce the probability of the off-targeting effect. In cases of gene knock-out, sgRNAs must be designed at a region that is common for all isoforms of a single gene [46]. Additionally, the cleaved site would be located within the exonic region or the splice site to have a complete loss of function [47,48]. This design can also be performed on functional domains of the protein, leading to an efficient inactivation of the protein [49]. Interestingly, once a non-functional RNA is created, the nonsense-mediated decay system cannot degrade it in RNA viruses [50,51]. In designing the sgRNA for viruses, the sequence should be selected with caution. In some cases, the beginning of one gene may be located within another gene; therefore, this region should not be considered the region for designing the sgRNA. Furthermore, different regions of a gene may have different functions; therefore, the design should be done carefully for the target region. For example, in the previous studies, sgRNAs were selected in the UL39 gene (ribonucleotide-diphosphate reductase large subunit) of the HSV-1 in a way that necroptosis- and apoptosis-inhibiting domains remained intact and completely functional, while the function of the viral ribonucleotide reductase subunit one protein, the gene that provides a DNA synthesis precursor, was lost [52]. If an sgRNA is designed downstream of the transcription start site during sgRNA design in CRISPRa/i, the polymerase function would be blocked, and the expression will be reduced [26,53]. Additionally, the use of several sgRNAs would help facilitate the better function and prevention of the sgRNA site block that would result in more effective transcriptional level changes [54]. GC count is also known as an important factor. In designing the sgRNA for a region of a viral genome with a high GC count, the design should be closest to the optimum GC count (40–70%) [55]. The percentage of GC counts in the 20-nucleotide sequence complementary to the sgRNA target region, if more or less than 40–70%, may affect activity, efficiency, accuracy, and the off-targeting effect. The next step (or another point to consider) is the secondary structure in sgRNA. The design should be with the lowest energy and the minimum probability of stem-loop formation because the presence of stem-loop or forming structures in sgRNA can significantly disrupt system functionality [56]. Studies have shown that the presence of specific nucleotides at specific sgRNA sites might affect system performance. It has been shown that the presence of adenine and guanine after the PAM sequence increases activity, but thymidine and cytosine act in an opposite way [57]. Additionally, it is important to know the type of sgRNA promoter. For example, when using the U6 promoter (a housekeeping gene promoter), the first transcription initiator nucleotide must be guanine to transcribe with high efficiency and high performance of the system [58]. In the case of using the T7 promoter, the beginning nucleotides of transcription should be GG to have a better function [59] (Figure 2).

Many software packages are introduced to facilitate sgRNA design by incorporating many of these factors and offering predicted functional sgRNAs (Table 2).

## 3. Essentials of Gene Knock-Out

Multiple sgRNAs can be used to target a gene to increase the efficiency of gene knock-outs [67,68]. The separate plasmids can be used for the expression of different sgRNAs (i.e., each vector expresses one type of sgRNA) [7]. In this case, the system’s efficiency is significantly reduced because each cell might have received different plasmids and, consequently, different sgRNAs. To overcome this problem, all of the sgRNAs are tandem repeat cloned using a single plasmid with sequences in between sgRNAs. These separating sequences could then be cleaved by the nuclease enzyme. Then, sgRNAs will be cut/separated as results significantly improved performance (for example, by using tRNA sequences between sgRNAs, these transcripts are naturally cleaved after splicing, and the sgRNAs are released) [69]. The functionality of a sgRNA can be tested in vitro through recombinant Cas9 protein and its related sgRNAs using the plasmid or polymerase chain reaction (PCR) product containing the target fragment. Upon successful/efficient cleavage, these sgRNAs can be used in vivo [70]. However, it should be noted that functioning in vitro does not guarantee the functionality of sgRNA in vivo for two reasons. First, if the target of a sgRNA is on cellular DNA, it may lose its function because the DNA molecule is packaged into structures called chromosomes or minichromosomes [71]. Second, intracellular conditions are significantly different from the in vitro environment. A well-performing sgRNA in vitro may not cut properly in vivo. The heterochromatin of targeted regions embedded in viruses may be a reason for the possibility that the CRISPR system does not work in cell-based assays. In the HSV-1 virus, 58sgRNA was designed for the RSI/ICP4 (gene expression regulator), UL54/ICP27 (transcription regulatory), UL30 (DNA polymerase catalytic subunit), and UL29 (major DNA-binding protein) genes. The capability to knock out these genes was first confirmed with spCas9 recombinant protein in vitro and was followed by the selection of functional sgRNAs. Functional sgRNAs were shown to be capable of reducing the viral titer from 1000- to 10,000-fold by a multiplicity of infection (MOI) of 5 and 0.01, respectively. Analysis of the functional regions of the CRISPR system by Miseq revealed that 99% of the indel mutations were equal to or less than 6 nucleotides [70].

Notably, 2 plasmids, one with full-length sgRNA (20 nt) and the other with sgRNA ≤15 nucleotides in length (instead of 20 nucleotides complementary to the target site), can be used to modulate gene expression and knock out genes simultaneously. Although Cas9 is shown to be able to bind specifically to DNA under such conditions (≤15 nucleotides), it is not capable of cutting. In this method, Cas9 binds to regulator module (i.e., transcription activators, suppressors, epigenetic modulators (DNA methyltransferase, acetyltransferases, deacetylases))-targeted sgRNA complementary regions to change gene expression and create a double-strand break in the other region [72,73]. Elimination of HIV-1 infection showed that Cas12a has an outstanding function compared to Cas9 due to a single sgRNA resulting in complete inhibition of HIV-1 infection in SupT1 cells [74]. It was also found that post-incision repair by Cas12a is distinct from Cas9 (Cas12a is not capable of producing the pure DNA insertion that occurs using Cas9) [74]. SaCas9 has also been shown to inhibit HIV-1 infection well in Jurkat C11, TZM b1, and Jurkat T cells [75]. Localized DNA fragment deletion is another method that can be used to manipulate the viral genome. Viruses with a size limit for packaging can be shrunk down by using sgRNAs on either side of an unnecessary region (by deleting that unnecessary region using the CRISPR system) and subsequently inserting the fragment of interest into the viral genome. Due to the ability to simultaneously target multiple regions in a genome or multiplexing, the CRISPR system can be used to make simultaneous changes in different regions and genes. Researchers have used this system to create a method called genome-scale CRISPR/Cas9 knock-out (GeCKO). This system is used to identify essential genes and is designed for all human genes through which extensive gene KO in the cell can be generated [76]. This method has been developed and introduced by modifying the sgRNA target regions (adding noncoding RNAs and a number of sgRNAs targeting human genes and the sgRNA control group) and has been introduced as GeCKO v.2 [77]. The system has been used to identify new immune cells acting against cancer cells and to detect the monomorphic MHC (major histocompatibility complex) class I-related protein MR1 receptor on cells [78]. Various examples of viral load reduction in different viruses are given in Table 3, and essentials for knock-out design are provided in Figure 2.

## 4. Knock-Out Detection Methods

PCR is one of the most common KO identification methods. It is designed with two primers on the top and bottom of the KO region. In this method, sgRNAs are designed to be located 20–30 nucleotides apart from each other. The deletion of 20–30 nucleotides from the PCR product of the sgRNA target can be detected by agarose gel electrophoresis [91].

Restriction fragment length polymorphism (RFLP) can also be used for KO detection. In this method, an indel mutation created by the NHEJ repair and the position where the restriction enzyme cutting site is located will be destroyed. The sgRNA is designed for the position where the restriction enzyme cutting site is located. If the CRISPR system’s designed section is cut and repaired with NHEJ, the enzyme cutting site is destroyed [92]. One of the most commonly used identification methods is the T7 endonuclease I or SURVEYOR enzyme. These methods enable the detection of heteroduplex DNA mismatches. When the DNA fragments in which the indel mutation has occurred combine with the intact DNA fragments, a heteroduplex is formed. T7E1 and SURVEYOR enzymes can cut these fragments. This cut can be detected on the agarose gel [93,94]. Another method of indel mutation detection is using a high-resolution melting curve (HRM), which can be used to identify heteroduplex DNA mismatches. First, the DNA fragment of interest is amplified using PCR. Then, special saturation dyes (called intercalating dyes) attach to the double-strand DNA (which is already in the reaction). First, the temperature increases to 50–95 °C, and then the temperature decreases very slowly to the point where there are nucleotide changes and the two strands of DNA are connected more quickly. This can be observed in real-time PCR instruments. Using this method, the SNP and indel mutations can be detected [95]. Various next-generation sequencing (NGS) platforms can also be utilized for this purpose, and several platforms have been developed to decrease its cost and enhance its accuracy and high-throughput NGS functionality [96,97,98]. Nevertheless, <0.5% of the off-target mutations are not detectable (sequencing error rate), and different methods are being developed to overcome this shortcoming [99]. Despite that, the current gold standard for indel mutations is Sanger sequencing [100,101].

## 5. The Perspective of CRISPR Tools in Virus-Cell Interactions

Extensive research has been conducted on CRISPR’s use in manipulating various viral RNA and DNA viruses and inhibiting the replication of viruses. Studies on the use of nickase and nuclease inactive-Cas9 systems to target HBc protein-coding gene (hepatitis B core/capsid protein) for inhibition of hepatitis B virus (HBV) replication in vitro (HepG2.2.15.7, HepG2-hNTCP-C4, and Huh7 cells) and in vivo (mouse liver) have shown the efficient elimination of viral infection. Furthermore, nickase and nuclease inactive-Cas9 systems reduced the simultaneous off-targeting effect in the host genome significantly [102].

CRISPR activation (CRISPRa) and CRISPR inhibition (CRISPRi) are subtypes of the CRISPR system. In these systems, changes have been made to cause the loss of the ability of Cas9 protein to cut double-strand DNA (dead Cas9 (dCas9)). By the binding of dCas9 to various functional domains (such as transcriptional inhibitors and activators (CRISPR I & a)) of protein Cas9, the ability to activate or inhibit gene expression by this system has been achieved. By using CRISPRa, the IFI16 (interferon-gamma-inducible protein 16) and IFNL2 (interferon-lambda 2) genes are activated. These two genes encode for proteins involved in the immune response and are shown to provide high protection against the Zika virus (in Huh7 hepatoma cells). Moreover, genome-wide overexpression screens have provided the possibility of identifying host factors associated with the virus that can be used as targets for drug development [103]. For instance, B4GALNT2 (beta-1,4-N-acetyl-galactosaminyltransferase 2) genes were identified as a factor against the pan-avian influenza virus in the host genome. Its increased expression has resulted in a 100-fold decrease in the virus titer (the study was performed on human lung epithelial cell line A549 and Madin–Darby canine kidney (MDCK) cells) [104]. In another study, it was shown that the downregulation of protein Moloney leukemia virus 10 (an RNA helicase) increased HBV DNA levels, but its overexpression reduced HBV DNA levels in HepAD38 (an inducible hepatoblastoma cell line with tetracycline (tet)-off-regulated CMV promoter for HBV infectious clones and HepG2 cells). This finding highlights the importance of this protein in viral production. This experiment identified this protein as an HBV antagonist [105].

It has been shown that the activation of endogenous BST-2/tetherin expression, a component for the innate immune response to enveloped viruses, using CRISPRa (VP64 (a viral transcription activator)) results in the inhibition of HIV-1 proliferation [106]. Additionally, the demethylation of the downstream of the IFN-β (interferon-beta) gene, one of the main factors in the immune system response, transcription start site in 1A5 and 28E10 cells has been shown to result in the inhibition of viral replication, as the viral titer was decreased 80 to 90% 72 h post-infection [107].

One of the main reasons for the resistance of HIV to treatment is the latency in resting CD4^+^ memory T cells [108]. Several studies have been performed using CRISPR systems to confirm the HIV-1 reversal of latency. In these studies, the synergistic activation mediator (SAM) activator was shown to perform better in the induction of HIV-1 latency. The SAM activator is a manipulated protein with an activation domain of HSF1 protein (heat shock factor 1) and NF-κB transactivating subunit protein 5 for the transactivating targeted gene(s). NF-κB is also known to be capable of recruiting histone acetyltransferases and thereby disrupting chromatin structures. This process most probably activates the imitation of transcription. Studies have also shown a significantly increased elongation in transcription after the addition of phosphor 65 (p65) to the histone deacetylase inhibitor trichostatin A-treated cells. This effect on elongation increase was similar to that of Tat (tyrosine aminotransferase). This evidence suggests that elongation is also further stimulated (or increased) by p65 without any effect on histone acetylation [109]. Additionally, this system’s performance in genomic transcription activation is specific, functional, and reversible with potential clinical applicability [53,54,110,111]. The use of CRISPR interference (CRISPRi) in CD4^+^ T cells has also shown that ELL2 (elongation factor for RNA polymerase II 2) plays a key role in regulating HIV latency. Elevation in the level of ELL2 and ELL2-SECs (super elongation complex) following proteasome inhibition is indicative of the proteasome-ELL2 axis’s important role in regulating HIV-1 latency, making it a promising target for therapeutic intervention [112]. In a study using the CRISPR system, 121 host genes associated with influenza A virus replication were identified, and WDR7 (WD repeat-containing protein 7, which is involved in gene regulation, signal transduction, cell cycle progression, and apoptosis), TMEM199 (transmembrane protein 199, with a role in protein localization in some cells), and CCDC115 (coiled-coil domain-containing protein 115, which is required for intracellular iron homeostasis in aerobic conditions and is a component of the proton-transporting vacuolar) host factors were shown to be essential for the entry and functioning of the virus. It was also demonstrated that the CMTR7 gene (a human mRNA cap methyltransferase) is associated with cell autonomic immune response [113]. Another study designed 8964 different sgRNAs to target and screen for vaccinia virus-specific genes. This study describes this system’s potential for simultaneous changes in other regions of the virus genome [114]. CRISPR knock-out was performed to find significant host factors in influenza virus replication in cells, and as a result of this study, it was shown that IFIT2 (an interferon-stimulated gene), which has antiviral activity, reduces infection [115]. A study of a CRISPR-wide SARS-CoV-2 genome screen showed that the SWI/SNF chromatin remodeling complex (a nucleosome remodeling mechanism), the TGF-β (transforming growth factor-beta) signaling pathway (with a role in many cellular functions), alarmin (which has a role in many cellular processes for example in gene expression regulation), HMGB1 (high mobility group protein B1) and H3.3 chaperone complex are the main factors related to the pathogenicity of this virus [116]. Genome-wide loss of CRISPR/Cas9’s screen function in human lung epithelial cells showed that SRPK1/2 (the virus nucleocapsid protein phosphorylase 1/2) is essential for SARS-CoV-2 replication. As a result, the inhibition of this gene reduced virus replication by more than 100,000-fold. Subsequent studies have shown that this protein phosphorylates the virus nucleocapsid in well-protected sites. This finding indicates that SRPK1/2 can be considered an effective therapeutic target [117].

The described system is one of the most functional systems for recognizing the host-virus interaction. Therefore, it is used to identify the molecular pathways associated with pathogenesis and find cellular genes/proteins as candidates for medicinal purposes. These studies show the vital role of host factors in causing viral infections. Genome-wide CRISPR–Cas9 screens show that the histidine methyltransferase SET domain containing 3 (SETD3) is a crucial factor in enteroviruses in multiple mouse model studies and have provided evidence that hepatitis A virus (HAV) and HBV are both co-dependent on the yeast Trf4/5–Air1/2–Mtr4 polyadenylation (TRAMP)-like complex despite distinct host factors. This is explained by (1) the dependency of the HAV translation on ubiquitin fold modifier 1 (UFM1)’s conjugation of the ribosomal protein RPL26 and (2) the necessity of components related to the TRAMP complex for viral translation independent of controlling HAV poly(A) tails. Furthermore, decreases in HAV replication have been shown in different cell types (i.e., hepatocyte cells and human liver organoids) followed by TRAMP-like complex inhibition through pharmacological approaches.

Principles for knock-in design (KI) is one of the most applicable techniques that have been simplified using CRISPR. By using this technique, we can insert target genes, nucleotide fragments, and target sequences to the targeted sites [118]. This method is used to add a peptide tag (i.e., poly-histidine tag (His tag), glutathione-S-transferase (GST), maltose-binding protein (MBP)) to a specific protein for the purification and identification of those that cannot have antibodies designed for them [119]. To determine the genes inserted by KI, a reporter gene (such as RFP (red fluorescent protein) or GFP (green fluorescent protein)) can be inserted into the target region, followed by different isolation techniques [120]. Virology research can utilize this method to detect KI in viruses. It has been proposed to insert a reporter gene for KO and KI genes in the viral genome and use it to purify the modified viruses. Isolation of KO and KI viruses can be easily and quickly done by this method.

## 6. Knock-In Efficiency Enhancement Strategies

The first point in enhancing KI efficiency is the ability to cut through the CRISPR system, which is directly related to the design and functionality of sgRNAs. One way to increase this capability is to use a maximum distance of 100 nucleotides between the left and right flanks and design multiple sgRNAs in this region. However, the distance between two flanks of left and right from the Cas9 cut site should be ten nucleotides, as it will increase KI efficiency [121].

Increasing the length of homology flanks to more than 1 kb significantly increases the KI efficiency [122]. In a study, 293A cells were infected with MOI 0.01 HSV strain KOS (multiplicity of infection), and viruses were harvested after 3 days. It was shown that recombination was not performed in cells transfected with less than 20% efficiency. For KI in the UL3 (coding an uncharacterized protein)/UL4 (coding envelope glycoprotein) intergenic region, flanks were designed with lengths of 0.5, 1, 2, or 3 kb. It was shown that KI was directly related to flank length (with about 10-fold efficiency) [123]. However, depending on the length of the inserted fragment, flank length can be increased.

Optimizing homologous recombination (HR) construct concentration relative to the plasmid containing sgRNA and Cas9 can contribute to the insertion efficiency; the best results are shown in a study where the proportions of five-folds of the HR construct to one-fold of the plasmid containing sgRNA and Cas9 was used. Additionally, making linear plasmids flanked by single-stranded DNA has been shown to improve KI efficacy. Nevertheless, it is shown that in the HSV-1 strain KOS, modifying the concentration of the plasmid-encoding CRISPR system and HR donor has a moderate effect on KI efficiency [123].

Even though NHEJ occurs at all cellular stages, HDR is higher in the late S and G2 phases [124]. KI efficacy can also be increased by using different drugs with other mechanisms of action such as RS1 (facilitators of homologous recombination-binding protein (RAD51) binding to the cleaved DNA region), Ku-0060648, and nu7441 molecules (NHEJ inhibitors, that result in higher HDR repair rates) and nocodazole (cell cycle arresters in G2 and M phases). The combination of NHEJ inhibitory (such as SCR7 (DNA ligase IV inhibitor)) and HDR-promoting factors (such as Rad57 (a DNA repair protein)) with Cas9 will also increase the rate of HDR [125]. ssODN (single-stranded donor oligonucleotides) with 200 bp length and 3 stop codons have been used to investigate the effect of SCR7 on KI efficacy in human cytomegalovirus and primary fibroblasts, which has resulted in 80–90% gene knock-out. Subsequent studies showed that with the use of the NHEJ repair system, the resulting indel mutation was responsible for the KO formation; cells treated with SRC7 and the use of ssODN did not improve the HR efficacy. In this study, KI efficiency increased about 17% by transfection through electroporation, the optimization of electroporation pulses, and the HR template’s concentration [122].

Furthermore, in another study, pre-incubation of HSV-GFP with SCR7 in MOI:1 inhibited NHEJ, and 1 µM concentration of SCR7 increased HR efficacy by more than 10-fold. Nonetheless, higher concentration did not affect the ineffectiveness. This study successfully inserted GFP into 2 copies of the ICP0 gene with about 0.85% efficiency [126]. The inhibition of promoters of Ku 70 and DNA ligase IV (with SRC7 inhibitor) increases HDR efficiency to 4-5-fold. Additionally, adenovirus E1B55K and E4orf6 proteins (E3 ubiquitin ligase complex) coexpression increase HDR 8-fold by ubiquitination and proteasomal degradation of DNA ligase IV reducing NHEJ [127]. By using CRISPRi techniques, it is possible to increase or decrease the expression of HDR-related efficiency factors and increase HDR.

MOI as a proportion of virus number to cell number also contributes to higher usage of HDR or NHEJ. It has been shown that by using a MOI of 5, HDR will be more efficient in HSV-1, and NHEJ will be effective when used at lower MOI. It is suggested to use MOIs of 0.01, 0.1, 1, and 5 to generate KI & KO viruses to achieve the best results. The results of the study on human cytomegalovirus (HCMV) showed that the NHEJ repair system acts quickly following the infection. Therefore, the probability of HR in the target region harboring the indel mutation is reduced [122]. It was shown that the highest cutting efficiency is in an MOI of 0.1 and 1 HSV-1 and reaches a maximum after 36 h of infection [126]. The most comprehensive study on HSV-1 has shown that the ICP0 gene affects DNA damage repair or chromatin modifications and virus genome packing. Thus, the probability of HDR occurring at a MOI of 5 is higher [70]. Alternatively, the DNA sequence containing GFP-blasticidin deaminase fusion protein was performed with homologous insertion site flanks in genomic DNA for KI in HCMV. In this method, different MOIs were used, and the maximum efficiency of 11.8 was reached at an MOI of 0.25 [122].

Single or double-strand donor oligonucleotides are used primarily to induce single or multi-nucleotide changes. For insertion with this method, flanks on both sides of the cleavage region are used for proper performance. The maximum length of this oligonucleotide fragment is 200 nucleotides, and the length of each of the right and left flanks is 50 to 100 nucleotides, such that each flank can be up to 7 nucleotides away from the cleavage site [128]. Additionally, the use of asymmetric or symmetric flanks can affect KI efficiency [129,130]. In several studies in which the fragment insertion is performed adopting this method, the knock-in efficiency with asymmetric or symmetric right and left flanks has been different [129,131]. It seems that comparative studies in each virus are needed to determine this method’s efficacy in viral studies. In a study designing a CRISPR/Cas9 system and a 138 nucleotide-length ssODN between the gE signal sequence and extracellular domain, 10% KI efficacy in the HSV-1 virus has been approved. TK encodes the thymidine kinase enzyme KO with a 50% efficacy. It indicates that this system is functional in viruses with large DNA genomes (e.g., cytomegalovirus, vaccinia virus, Epstein–Barr virus, varicella-zoster virus, etc.) [132]. Another way to prove its effectiveness and performance in viruses is a design by which the 2 inserted fragment ends are 100 bp in single-stranded flanks. This method has shown high efficiency for the creation of KI [133].

By linking different deaminases to Cas9, precise single-nucleotide changes can be made on the target region. It has been shown that this method is efficient and straightforward, and with the Cas9 D10A double nickase method, the generation of an acceptable percentage of KI has been successful. In a study, the pseudorabies viral genome was cloned into the bacterial artificial chromosome (BAC). Then, it was edited by the CRISPR/Cas9 cytidine deaminase fused protein system, which resulted in turning cytidine into uridine with 100% efficacy, along with the creation of a stop codon in the frame [33]. The methods described above can increase HDR efficiency and consequently increase insertion by more than 30 folds relative to the regular cellular rate (Figure 2).

Non-cleavage genome-editing single base-pair editing systems are designed to make changes in the genome without DNA cleavage (resulting in reduced indel mutations and off-target effects) in two types of cytosine base editors and adenine base editors [134,135]. Different classes of base editors have been designed (BE1 (base editor 1), BE2 (base editor 2), and BE3 (base editor 3)) and can change a specific base using CRISPR technology. In H840 (histidine 840), the HNH BE3 domain has mutated to lose the ability to generate nickase. This system significantly improves the editing efficiency and changes the U-G to U-A (uracil-guanine to uracil-adenine) by creating a cut in the G-containing strand, which becomes T-A (thymine-adenine) during replication. However, this system increases the rate of indel mutations compared to BE2, which does not have the property of creating nickase (1.1% compared to 0.1%) [134,136]. Various BE systems are designed based on the Cas system’s different nucleases to identify more sites by Cas systems and to create BE in other locations. This system can be used to develop targeted point mutations in genes [137,138]. The streptococcus pyogenes-Cas9-base editor (Sp-Cas9-BE) effectively edited the surface genes and polymerases. As a result of this base editing, episomal covalently closed circular DNA (cccDNA) was successfully edited, and HBV gene expression was decreased with very low induction of indel mutations [139]. In 2019, a smart design was implemented in the CRISPR system using expanded sgRNA-containing flanks, short insert sequences and a reverse transcriptase fused to Cas9. After Cas9 protein cleavage, single-stranded DNA acts as a primer. The enzyme synthesizes complementary DNA (cDNA) from the expanded sgRNA, which complements the cleaved region and has the modified region inserted. Then, the fragment inserts into the target DNA, and the DNA repair mechanism copies the complementary strand. Without inserting the insert fragment into the cells separately, KI was shown to perform with high efficiency. Using this method (called prime editing), any short nucleotide changes can be made. This method has the potential to be applied for making targeted changes in viruses [140].

## 7. RNA Editing

Cas13 (C2c2) is similar to bacterial-derived Cas9 and is a type of bacterial immunity to phages with the RNA genome [141]. Scientists have discovered several subtypes of this system, modified Cas13 by making changes to Cas13, and named these subtypes as Cas13a-d, as follows:

Cas13a: Derived from *Leptotrichia wadeii* (LwaCas13a) [142];

Cas13b: *Prevotella* sp. P5-125 (PspCas13b) [143], Cas13d: *Ruminococcus flavefaciens* (RfxCas13d) [144] bacteria which is capable of RNA cleavage. Changes to Cas13a have resulted in inactive Cas13a that has lost its ability to cleave and can only bind to RNA. The binding of modules on the inactive Cas13a has made it possible to make massive changes [142]. By linking the RNA adenosine deaminase protein module to the inactive Cas13, the ability of targeted A + I (G) conversion in RNA was achieved in Cas13b [143];

Cas13c: Recently identified, and its performance is under investigation [142];

Cas13d: It has very high efficiency and does not have the limitation of the protospacer flanking sequence (PFS) [144].

These new and functional systems can be used to modify and evaluate the function of RNA viruses, changes in the level of RNA and DNA viruses, and cellular changes to understand the function of viruses. Extensive research on RNA (viral and non-viral) function can also be done using inactive Cas13 attached to a reporter. Targeting the HPV 18/16 E6 gene by the CRISPR/Cas13a system in E6-transformed keratinocytes induces apoptosis and inhibits proliferation [145]. Cas13a, in addition to lowering the RNA level of the newly synthesized HIV-1 virus, destroys the RNA of viruses that enter the cell with a viral capsid and can strongly inhibit the HIV-1 virus in HEK 293T cells (3 h after infection) and Jurkat cells (3 days after infection) and RNA expression from latent HIV-1 DNA (in JLat10.6 HIV latent cells) [146]. Cas13a successfully reduces the expression of the HIV-1 virus gene (in normal and latent HIV-1 infected cells) at the early and late stages of infection with high efficiency in destroying viral RNA. It significantly inhibits infection in HEK293T, Jurkat, and Jlat10.6 cells [146]. EBT-101 is one of the in vivo therapeutic designed CRISPR technologies in the clinical trial (phase 1 & 2) (NCT05144386). In this clinical trial, large sections of HIV proviral DNA are cut with the CRISPR technology, and patients stay on long-term follow-up (NCT05143307]. Targeting the Dengue virus NS3 gene using Cas13 has resulted in the inhibition of virus replication [147]. CRISPR/Cas13d as an RNA targeting system in the conserved region has also been specified to have a very high potential for targeting SARS-CoV-2 and influenza genome (H1N1 live influenza A virus) as well as a reducing viral load in lung epithelial cells with the PAC-MAN system (prophylactic antiviral CRISPR in human cells) [148]. Furthermore, targeting the SARS-CoV-2 spike protein with Cas13a in HepG2 and AT2 cells has led to a robust decrease in viral load [149].

### 7.1. Diagnosis of Viral Infection

In the last few years, the ability to clinically detect viruses using the CRISPR technique has attracted increasing attention. This technique has created more straightforward, faster, easier, cheaper, and more accurate diagnostic platforms. One of the most critical advances in this field was made in 2019 with the design of a quick, precise, and highly sensitive system called SHERLOCK (specific high sensitivity enzymatic reporter unlocking) to detect the Zika virus by the CRISPR system. This system can also be used to identify RNA samples. The first step to use this system is to amplify RNA (RNA converts to DNA, then amplifies). Then, in less than 1 h, the DNA can be detected by colorimetric or fluorescence methods through the cleavage of a specific DNA region by Cas13a targeted endonuclease. It results in the quencher being separated from the reporter, from which we can identify the reporter. The basis of all these identification techniques derived from the CRISPR system is relatively identical. It is based on specific sequences unique to the virus or organism targeted by Cas nuclease. Another technology is DETECTR (DNA endonuclease-targeted CRISPR trans reporter). In this technology, by using Cas12a, one can detect DNA samples the same way as with SHERLOCK technology. For instance, these techniques have been used to detect avian influenza A (H7N9), Ebola, Epstein–Barr virus, and African swine fever viruses [150,151]. Extensive studies have been conducted on SARS-CoV-2 detection by using the CRISPR system. These studies have led to the development of viral molecular detection kits using Cas13, as well as Cas12 endonuclease proteins, which have a high sensitivity (ability to detect a few copies) and specificity, low cost, and high-speed detection. This technique has introduced a new generation of molecular and market diagnoses [152,153,154,155,156,157]. Thus far, one clinical trial of the CRISPR-based SARS-CoV-2 detection kit is finished (NCT05107258]), and the other one is in the recruiting phase (NCT05034978).

### 7.2. CRISPR Delivery Systems

There are many methods for delivering the CRISPR system to target tissues and cells based on two approaches of viral transmission (AAV, adenovirus, and lentivirus) and non-viral transmission (liposomes, electroporation, microinjection, and nanoparticles) [158,159,160]. Adeno-associated virus is known as the leading delivery for viral transmission of genome editing agents [161]. This virus has double-stranded DNA and can pass through the blood–brain barrier. It can transduce in a broad range of dividing and non-dividing cells [162,163]. Thus, this viral vector can be used to target viruses that have targets in the brain or are latent in the brain (such as HSV-1). Several clinical trials are using the AAV virus, some of which are FDA-approved (Zolgensma for spinal muscular atrophy and Luxturna for a rare inherited retinal dystrophy) [161]. However, one of the problems of the AAV virus is the reaction of host immune response, which can inhibit this virus [161,164]. Despite the fact that another limitation is its low vector capacity (less than 5 kb) [165], the adenovirus can accept 8.5 kb of DNA [166]. Some types of these viruses have reached an acceptance capacity of about 37 kb (helper-dependent adenoviral vectors) by removing viral genes [167]. Limitations of this viral vector include a pre-existing immune response, restriction of tissues without adenoviral receptors, and liver sequestration [168]. Adenovirus DNA cannot integrate into DNA but can remain episodic. It has been shown that replication-incompetent adenoviral vectors can integrate into infected cells with a frequency of 0.001–1% [169]. Lentiviruses are manipulated wild viruses that have lost their pathogenicity and have a high affinity for transmitting genetic material. This virus is derived from HIV-1, can integrate randomly into the DNA, and can create insertional mutations. Lentiviral vectors could stabilize gene expression in the dividing and nondividing cells and package about 9 kb of single-stranded RNA. This system is widely used in virologic research using the CRISPR system [170,171,172,173].

Liposomes are also one of the most widely used systems for delivering genetic material to cells. The basis of all liposomes is the encapsulation of genetic material by lipids and liposomes’ integration with cell membranes. For a better and more efficient delivery through this transmitter of genetic materials, various types of liposomes have been designed (e.g., lipiodol nanoparticles (lipid-like molecules derived from PEG (polyethylene glycol), modified lipids and cholesterol) and cationic liposomes (positively charged lipid structures for nucleic acid uptake and better binding to cell membranes)) [174]. This system has a high cost and less stability [175]. Liposome delivery of CRISPR system against HPV 18 E6 and E7 genes in mice xenografts and injection into the tumor effectively inhibited tumor growth [176].

Electroporation is another method that temporarily creates micropores in cell membranes that lead to the absorption of proteins and genetic material. This system is efficient and has a very low cost. It also requires a minimal DNA concentration and works with a broad range of cell types. However, electroporation has the potential to damage cells and may transport nonspecific molecules (using the CRISPR/dCas9 fusion VP64 domain system, HIV-1 virus latency was well activated in cells) [110,177,178].

Microinjection is one of the best methods, with a delivery capability of nearly 100% and without loading capacity (it consists of a glass micropipette (to fix the zygote) and needle that can transfer genetic material directly into the zygote). However, this system should be optimized because it is not high throughput and two microinjections in a few hours lead to cell inactivation. Nevertheless, this technique is known as the gold standard for delivering the CRISPR system to a zygote [168,179].

Potential nanoparticles also have the conductivity of the CRISPR system. These nanoparticles include gold nanoparticles and cationic nanoparticles [53,180]. For example, using gold nanoclusters, Cas9 proteins were introduced into cells and knocked out the E6 oncogene. This function induced apoptosis in human cervical cancer HeLa cells (which contain integrated HPV18 DNA and express the E6 gene) and had little effect on normal human cells (HEK-293T) [180].

### 7.3. CRISPR Ethics in Virology

CRISPR has evolved much faster than other genome editing techniques (TALEN and ZFN). Although it is faster, cheaper, more accessible, and more efficient [181], this system has limitations that have restricted its clinical application. These include restrictions in on-target efficiency, being off-target [182,183], the ability to inherit changes made to the next generation, the possibility of mosaics being created by this system, and the irreversibility of many changes [184]. Given its high potential for modifying the genome of living organisms and the potential to improve and treat many diseases [181], one of the most critical concerns could be irreversible harmful changes caused to the viruses. This system has a high potential to change organisms’ genomes for improving and treating viral infections and making beneficial changes to these viruses (e.g., oncolytic viruses) [52,185]. In the meantime, there is a potential threat of harmful and irreversible changes made to the viruses, leading to the extinction of species and the widespread death of various creatures. Although there are many concerns about using this system in living organisms discussed at numerous conferences, there seems to be a universal consensus and law to limit the use of this technique merely to make useful changes [186,187].

## 8. Conclusions

Researchers have been making changes to viruses for years to achieve different therapeutic purposes, such as antiviral therapies and oncolytic viruses [114,188,189]. However, these modifications are difficult, time-consuming, and costly [190]. As discussed in this article, CRISPR technology, as the most advanced and widely used genome editing technique, has made it much easier, cheaper, and more efficient to make changes to the viral genome. This technique enables the KI and KO gene activation/inhibition and epigenetic changes in the genome in one or more genes [191,192,193,194,195]. CRISPR has also led to many advances in the field of virology. For example, compared to the shock and kill strategy for HIV-1 reactivation and latency that has some disadvantages, the CRISPR system has been shown to perform satisfactorily without any pitfalls [196,197,198].

This technique can eliminate viral infection and make changes to viruses in vivo. Therefore, many advances in the diagnosis and treatment of viral infections have been achieved by the CRISPR technology, demonstrating its clinical potential in rapid virus detection, the reduction of viral load, and even the elimination of viral infections [199,200,201,202,203,204,205,206,207,208,209,210,211]. However, the highly effective application of viral genome changes requires precise and well-designed optimization of the conditions for targeted changes in the viral and host genomes.

It should be noted that off-target attachment to vital locations can lead to cell damage and result in apoptosis or shifting of normal cells towards cancer [206,207]. Moreover, the delivery of this system in vivo to the site of infection by viral vectors can lead to immunotoxicity against the Cas9 protein [200]. However, in the setting of virus manipulation in vitro, this problem is completely solved.

Further studies are necessary to overcome the remaining shortcomings of this technology.

## Figures and Tables

**Table 2:** List of software tools available for CRISPR sgRNA design.

Online Software	PAM	Nuclease	On- and Off-Target Prediction	CRISPR a and I Prediction	URL
CRISPRscan	NGG TTTV TTTN	Cas9 Lb Cas12a AsCas12a	Yes	No	https://www.crisprscan.org [57,60]
Cas-OFFinder	Broad PAMs	Broad species Cas9 and Cas12 nucleases	Yes	No (ability to base edit design)	http://www.rgenome.net [61]
CHOPCHOP v3	Variable PAM	Cas9 Cas12 Cas13	Yes	Yes	https://chopchop.cbu.uib.no [62]
CRISPOR	Variable PAM	Cas9 Cas12	Yes	No	http://crispor.tefor.net [63]
GuideScan	NGG TTTV TTTN	spCas9 AsCas12a LbCas12a	Yes	No	http://www.guidescan.com [64]
WGE	NGG	spCas9	Yes	No	https://wge.stemcell.sanger.ac.uk [65]
E-CRISP	NGG(NAG)	spCas9	Yes	No	http://www.e-crisp.org/E-CRISP/ [66]

**Table 3:** Examples of viral load reduction by using the CRISPR toolkit.

Virus	Gene(s) or Region	Strategy	The Decrease in Virus Load
HSV-1	UL52 and UL29	Cleavage	Complete suppression of HSV-1 infection within two days in Vero cells [79]
HPVs	E6	Cleavage	Reduced expression of E6 in Hela, HCS-2, and SKG-I cells and a mouse model of cervical cancer and, as a result, increased expression of p53; as a result, it is affecting in treating patients with cervical cancer [80]
HPV18	E6 and E7	CRISPRi	Inhibition of expression of E6 and E7 in Hela cells [81]
HBV	Conserved regions of the HBV genome	Cleavage	Degradation of over 90% of HBV cccDNA by 6 days post-transfection [82]
EBV	Targets different regions of the EBV genome	Cleavage	Levels of EBV DNA in transfected cells were decreased by about 50% in C666-1 cells [83]
HIV-1	LTR	Cleavage	CRISPR/Cas9 system may be a useful tool for cutting HIV-1 infection [84]
Polyomavirus JC	Gene encoding T-antigen	Cleavage	Gene-editing strategy as a promising tool for the elimination of the JCV genome [85]
HSV-1	gD gene	Cleavage	Successful HSV-1 infection was reduced in the HEK293D cell line [86]
HCMV	IE genes	Cleavage	Cas9 inhibits virus replication and reactivation [87]
HBV	cccDNA [closed circular DNA)	Cleavage	Reduced viral replication in huh7 and HepG 2.2.15 cells followed by Cas9 targeted with 4sgRNA designed for HBV protected regions. Similar results were replicated in mouse models [88]
HBV	cccDNA	Cleavage	Elimination of HBV infection in the stable HBV cell after a 3.175 bp fragment deletion and ccDNA degradation through CRISPR system [89]
HBV	Conserved regions	Cleavage	Multiple inhibitions both in vivo (mouse) and in vitro (HepG2 cells) [90]

HSV-1: herpesviruses-1, HPVs: human papillomavirus, HPV18: human papillomavirus 18, HBV: hepatitis B virus, EBV: Epstein–Barr virus, HIV-1: human immunodeficiency virus-1, HCMV: human cytomegalovirus.
